# A bibliometric analysis of global research on vitamin D and reproductive health between 2012 and 2021: Learning from the past, planning for the future

**DOI:** 10.3389/fnut.2022.973332

**Published:** 2022-09-08

**Authors:** Yimeng Lu, Xudong Zhang, Shanshan Wu, Siwen Zhang, Jichun Tan

**Affiliations:** ^1^Center of Reproductive Medicine, Department of Obstetrics and Gynecology, Shengjing Hospital of China Medical University, Shenyang, China; ^2^Key Laboratory of Reproductive Dysfunction Disease and Fertility Remodeling of Liaoning Province, Shenyang, China

**Keywords:** vitamin D, vitamin D deficiency, pregnancy, bibliometric analysis, reproductive health, marker

## Abstract

**Background:**

Vitamin D plays an invaluable role in reproductive health, but vitamin D insufficiency and deficiency are generally common among couples of childbearing age and pregnant women. This study aimed to evaluate the evolution, development trend, and research hotspot of publications on vitamin D and reproductive health.

**Methods:**

The literature on vitamin D and reproductive health between 2012 and 2021 was retrieved from the Web of Science Core Collection (WoSCC). We used VOSviewer and CiteSpace to analyze publication years, countries, institutions, journals, highly cited authors and publications, and co-occurrence and citation bursts of keywords.

**Results:**

A total of 1,828 articles and reviews on vitamin D and reproductive health published between 2012 and 2021 were identified. The annual publication outputs showed steady growth, with the most publications (272) and citations (7,097) in 2021. The United States contributed the most publications (458) and had the highest *h*-index (58). In terms of the number of publications and *h*-index, the journal named Nutrients ranked first. Nutrition dietetics, obstetrics gynecology, and endocrinology metabolism were three well-represented disciplines in research on vitamin D and reproductive health. Hollis BW, Wagner CL, and Litonjua AA were the top three most productive authors in this field during the last decade. Apart from vitamin D, the five keywords with the most frequent occurrence were vitamin D deficiency, pregnancy, risk, vitamin D supplementation, and 25-hydroxyvitamin D. Keyword citation burst analysis revealed that low birth weight, adipose tissue, marker, and embryo had a citation burst lasting until 2021.

**Conclusion:**

In conclusion, vitamin D has received continuous attention in the field of reproductive health, and there appears to have a higher level of research in North America. Multidisciplinary intersection contributed to the in-depth exploration in this field. And the effect of maternal vitamin D levels on fetal lipid metabolism and the prediction of fertility by vitamin D-related markers might be hotspots for the research.

## Introduction

Vitamin D is an essential fat-soluble vitamin that serves a vital role in physiological processes such as calcium and phosphorus metabolism, immune regulation, cell growth, differentiation, and apoptosis in the body (1). Vitamin D is a steroid derivative, which can be divided into ergocalciferol (vitamin D_2_) and cholecalciferol (vitamin D_3_) according to the chemical constitution (2). Vitamin D_2_ is sourced from the UV irradiation of ergosterol in some plants or fungi, while vitamin D_3_ is mostly synthesized from 7-dehydrocholesterol in the skin following the UV irradiation (3). It is estimated that about 80% of the body’s vitamin D comes from the endogenous synthesis in the skin (ambient UV exposure), and only 20% comes from exogenous intake, including vitamin D_3_–exogenous (egg yolks and oily fish), vitamin D_2_ fortified foods (margarine and breakfast cereals) and vitamin supplements (3, 4). After binding to the multifunctional vitamin D-binding protein (VDBP), vitamin D is transported to the liver, where it is transformed by the action of 25-hydroxylation into 25-hydroxy vitamin D_3_ [25-(OH)D_3_], which is its main form of circulation and storage in the human body. Then, 25-(OH)D_3_ needs to be metabolized to 1,25-(OH)_2_D_3_, the main active form of vitamin D, by the action of 1α-hydroxylase, which is expressed by many cell types (i.e., skin, immune cells, bone cells, placenta), but the highest concentration is found in the kidney proximal tubule cells (5, 6).

1,25-(OH)_2_D_3_ exerts hormone-like effects by binding to the vitamin D receptor (VDR). VDR belongs to the steroid receptor family, widely present in various tissues of the body, including the intestines, renal tubules, skin, pancreas, skeleton, immune system, and germ tissues (7). The binding of 1,25-(OH)_2_D_3_ to the ligand-binding domain of VDR promotes the phosphorylation of VDR and the heterodimerization of retinoid X receptor (RXR) to form a 1,25-(OH)_2_D_3_-VDR/RXR complex which interacts with the vitamin D responsive elements (VDRE) in the promotor region of target genes to exert the genomic effects (6, 8). In the non-genomic pathway, the 1,25-(OH)_2_D_3_-VDR-caveolin-1 complex induces modifications in cellular signaling pathways, thereby affecting cellular function (9). The VDR gene, located on chromosome 12, has single nucleotide polymorphism, which can have biological functions (8). VDR polymorphisms are closely related to renal diseases, bone biology, cancer, diabetes, and other diseases, which are expected to be a diagnostic tool for the diseases, but further large-scale population studies are needed (10).

The high prevalence of vitamin D deficiency is a worldwide problem, especially in pregnant and reproductive-age women (11, 12). Recent studies have shown that vitamin D played an important role in the regulation of female reproductive health and was involved in the oocyte development, and production of anti-mullerian (AMH), ovarian steroidogenesis, and endometrial receptivity (13). Vitamin D deficiency can lead to adverse pregnancy outcomes and cause a variety of reproductive disorders. Some meta-analyses have reported that vitamin D deficiency could increase the risks of preeclampsia, gestational diabetes mellitus (GDM), maternal infections, intrauterine growth restriction, and preterm birth (14–16). Vitamin D deficiency is associated with a high risk of hypertensive disorders in pregnancy (17). Matias et al. found that vitamin D can downregulate the activation of the inflammasome and the TLR4-MyD88-NF-κB pathway in preeclampsia (18). Evidence suggested that vitamin D could improve insulin sensitivity by enhancing insulin responsiveness to glucose transport (19) and thus prevent the occurrence of GDM. A prospective study demonstrated follicular fluid vitamin D level was positively correlated with clinical pregnancy and implantation rates and was an independent predictor of the success of *in vitro* fertilization treatment (20). Moreover, vitamin D was also involved in the development of reproductive disorders including polycystic ovary syndrome (PCOS) (21), endometriosis (22), and uterine fibroids (23).

Bibliometric analysis is a world-accepted statistical evaluation of published articles and has grown in popularity (24), which was first proposed by American bibliographers in 1969. Through qualitative and quantitative analysis of publications, it could use literature metrology characteristics to provide investigators with crucial messages and discover frontiers and evaluate the distribution of countries/regions, authors, and journals in a certain specific field (25). In line with the growing interest in the role of vitamin D in reproductive health, there is a growing body of literature, including numerous systematic reviews and meta-analyses, but few bibliometric analyses explored the hot spots and frontiers of research in this field. In this study, we aimed to provide a general description of quantitative and visual information in the global literature on the associations between vitamin D and reproductive health, identifying its emerging trends and potential hot spots from various aspects through integrative analysis of relevant information from manuscripts published worldwide from 2012 to 2021. We presented a brief discussion of vitamin D-reproductive research and predicted possible trends in this field over the next few years, laying a foundation for the direction and development of future research.

## Materials and methods

### Data collection and search strategy

All relevant articles published between 2012 and 2021 were retrieved from the Web of Science (WoS) Core Collection (WoSCC), one of the most widely used literature search databases. The main merits of the WoS database are a wide range of applications, few utilization restrictions, convenience to support horizontal comparison, and robust tools that assist in conducting advanced assessments over gathered information. The search strategies were as follows: Topic = (“reproducti*” OR “*fertil*” OR “steril*” OR “pregnan*”) AND Topic = (“*Vitamin D*”) AND Language = (English). The timespan was set from January 1, 2012, to December 31, 2021. For document types, only original articles and reviews were included in the study, with a total of 4,187 publications. Similarly, we searched for studies with the Topic = (“Vitamin D*”) following the same search strategies. A total of 48,493 relevant literature were retrieved. To avoid possible bias produced by continuous database updating, the retrieval and export of documents were created within 1 day (May 1, 2022). As well, to ensure the accuracy of retrieval, two independent authors (Xudong Zhang and Yimeng Lu) performed the search process and confirmed the search query and results. After excluding those on unrelated search topics (*n* = 2,359), the remaining 1,828 publications were included in the subsequent analyses.

### Data extraction and analysis

The retrieved data were downloaded and exported into different formats for further analysis. The “Analyze Results” and “Citation Report” functions of WoS were utilized for basic information statistics, including annual production, WoS categories, and quality of publications by country, institution, author, and journal. The quality of publications in this study mainly referred to the number of publications, the sum of the times cited, average citations per item (ACI), and *h*-index. Impact factors and category quartiles of journals for individual publications were collected from the 2021 Journal Citation Reports (JCR) (Clarivate Analytics, Philadelphia, United States). The country distribution maps were created using the online mapchart.^1^

VOSviewer and CiteSpace, two common software for bibliometric analysis, were employed to further analyze the underlying connections and key themes. We used VOSviewer (1.6.17, Leiden University, the Netherlands) to visualize co-authorship among countries/institutions/authors, co-citation of references, and co-occurrence of keywords. The specific steps included creating a map based on the bibliographic data, reading data from bibliographic database files, selecting the appropriate type and unit of analysis, and setting the counting method to full counting. In the figures obtained, the node represented country/institution/author/keyword, the size of the node indicated the number of publications or frequency of occurrence, the thickness of the curve showed the strength of the link, and the color reflected the cluster. CiteSpace is a Java-based software program and one of its features is citation burst analysis (26). CiteSpace (5.8. R3) was utilized to view citation bursts and a timeline of keywords. The parameters of CiteSpace were set as follows: link retaining factor (LRF = 3), look back years (LBY = 5), time slicing (from 2012 to 2021), years per slice (1), links (strength = cosine; scope = within slices), selection criteria (g-index, *k* = 25), and minimum duration (MD = 2). In the network graph, nodes represented various keywords, whereas the size of nodes reflected the frequency, and the connections between nodes represented the link.

## Results

### Temporal distribution map of the literature

**FIGURE 1:**
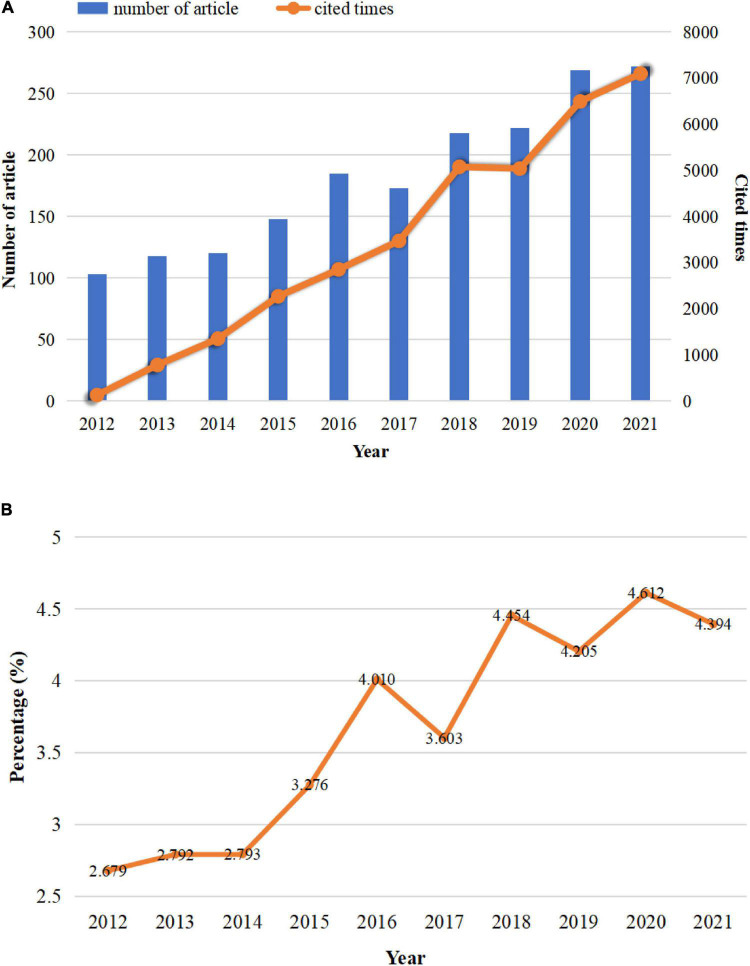
Trends in the growth of publications from 2012 to 2021. **(A)** The number of publications and citations per year on vitamin D and reproductive health. **(B)** Trends in the proportion of research on vitamin D and reproductive health in vitamin D-related research.

A total of 1,828 pieces of literature from 2012 to 2021 related to Vitamin D and reproductive health were retrieved from WoS. There were 1,534 articles (83.92%) and 294 reviews (16.08%), respectively. As shown in Figure 1A, the number of publications on vitamin D and reproductive health generally showed an upward trend. From 2012 to 2016, the number of publications increased steadily, with a slight decline in 2017, and then continued to increase until reaching its peak (272 publications) in 2021. Similarly, the percentage of research on vitamin D and reproductive health in all vitamin D-related publications gradually increased from 2012 to 2016, declined in 2017, and remained volatile at approximately 4.40% from 2018 to 2021 (Figure 1B). Cited times of publications increased year by year, except for a slight drop in 2019, and the most citations (7,097 times) were achieved in 2021 (Figure 1A).

### Distribution of countries and institutions

**FIGURE 2:**
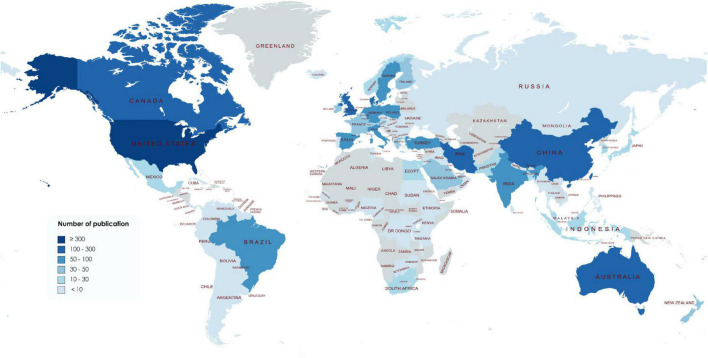
World map of countries contributing to vitamin D and reproductive health.

**FIGURE 3:**
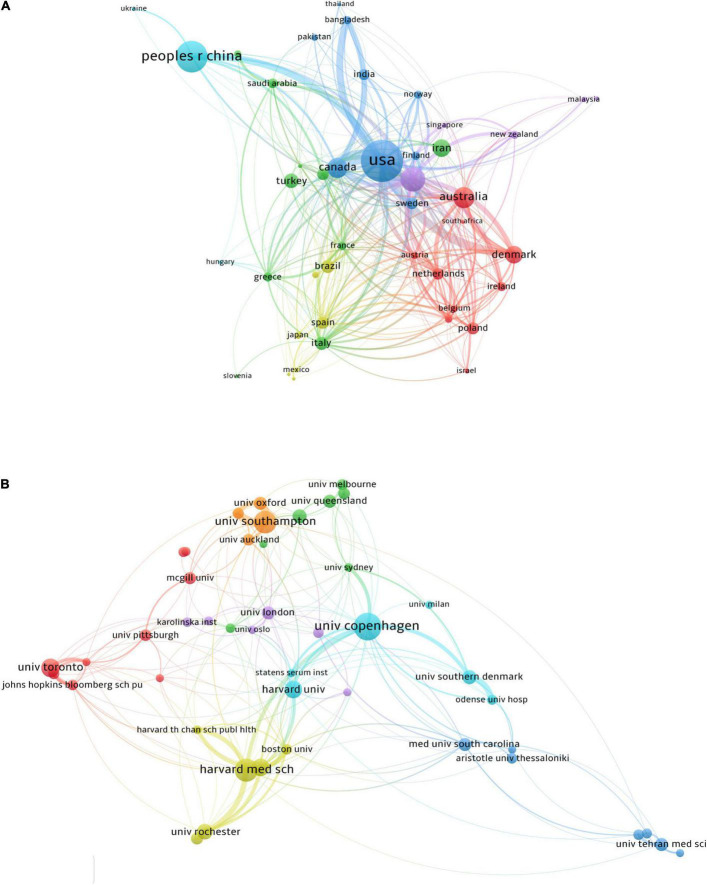
Co-authorship analysis of countries and institutions. **(A)** Network map of co-authorship between countries with more than five publications. **(B)** Network map of co-authorship between institutions with more than 15 publications.

A total of 96 countries contributed to publications in this field (Figure 2). As shown in Table 1, the United States contributed the greatest number of publications (458, 25.06% of all), followed by China (283, 15.48%), England (187, 10.23%), Australia (147, 8.04%), and Canada (139, 7.60%). The top five countries in terms of ACI values were Canada (29.00), the United States (28.91), England (27.78), Denmark (24.29), and Australia (21.61). Regarding the *h*-index, the United States (58), England (38), and Canada (35) ranked in the top three. A total of 44 countries with more than five publications in the field were analyzed in the co-authorship analysis (Figure 3A). The five countries with the highest total link strength were the United States (total link strength = 359 times), England (264), Canada (168), Australia (135), and Denmark (124) (Table 1).

**TABLE 1:** Top 10 productive countries regarding the research on vitamin D and reproductive health.

Rank	Country	Region	Quantity	Percentage	ACI	h	Total link strength
1	United States	North America	458	25.06%	28.91	58	359
2	China	Eastern Asia	283	15.48%	13.21	31	65
3	England	Western Europe	187	10.23%	27.78	38	264
4	Australia	Oceania	147	8.04%	21.61	32	135
5	Canada	North America	139	7.60%	29.00	35	168
6	Iran	Western Asia	107	5.85%	17.71	28	25
7	Denmark	Northern Europe	106	5.80%	24.29	30	124
8	Turkey	Western Asia	82	4.49%	8.76	14	20
9	Italy	Southern Europe	68	3.72%	19.87	23	109
10	Brazil	South America	66	3.61%	9.15	15	53

ACI, Average Citations per Item; h, h-index.

As shown in Table 2, the institution with the highest number of research publications in this field is Harvard University with a quantity of 96, followed by the University of Copenhagen (68) and Harvard Medical School (47). The research institution with the highest ACI value in this field was the Medical University of South Carolina, which had an ACI value of 65.62, followed by the University of London (31.72) and Brigham and Women’s Hospital (31.40). We analyzed the co-authorship of 46 institutions with more than 15 publications. The exclusion of one item that was not connected revealed the collaborations of 45 institutions (Figure 3B). The three institutions with the highest total link strength were the University of Copenhagen (total link strength = 149 times), Harvard Medical School (145), and Brigham and Women’s Hospital (127) (Table 2).

**TABLE 2:** Top 10 institutions in the studies of vitamin D and reproductive health.

Rank	Institution	Country	Quantity	STC	ACI	Total link strength
1	Harvard University	United States	96	2,884	30.04	95
2	University of Copenhagen	Denmark	68	1,601	23.54	149
3	Harvard Medical School	United States	47	1,031	21.94	145
4	Harvard T.H. Chan School of Public Health	United States	45	1,316	29.24	61
5	University of Southampton	England	45	1,351	30.02	126
6	University of Toronto	Canada	44	1,066	24.23	82
7	Brigham and Women’s Hospital	United States	43	1,350	31.40	127
8	University of London	England	43	1,364	31.72	63
9	Medical University of South Carolina	United States	42	2,756	65.62	43
10	National Institutes of Health	United States	41	792	19.32	55

STC, sum of the times cited; ACI, average citations per item.

### Analysis of journals and distribution of disciplines

A total of 1,828 articles were published in 518 journals. As shown in Table 3, the journal with the highest number of articles in this field was Nutrients (108), followed by PLoS One (68), Journal of Steroid Biochemistry and Molecular Biology (49), Journal of Maternal Fetal Neonatal Medicine (43), and BMC Pregnancy and Childbirth (42). The journal with the highest ACI value was Journal of Clinical Endocrinology Metabolism (41.69), followed by American Journal of Clinical Nutrition (34.78), Fertility and Sterility (34.68), PLoS One (22.46), and European Journal of Clinical Nutrition (21.95). The top five journals in terms of *h*-index were Nutrients (25), PLoS One (24), Journal of Clinical Endocrinology Metabolism (24), BMC Pregnancy and Childbirth (17), and American Journal of Clinical Nutrition (17).

**TABLE 3:** Top 15 journals in the studies of vitamin D and reproductive health.

Rank	Journal title	Quantity	ACI	IF	Q	h
1	Nutrients	108	18.64	6.706	Q1	25
2	PLoS One	68	22.46	3.752	Q2	24
3	Journal of Steroid Biochemistry and Molecular Biology	49	17.76	5.011	Q2	16
4	Journal of Maternal Fetal Neonatal Medicine	43	14.67	2.323	Q3	10
5	BMC Pregnancy and Childbirth	42	17.79	3.105	Q2	17
6	Journal of Clinical Endocrinology Metabolism	35	41.69	6.134	Q1	24
7	British Journal of Nutrition	34	19.59	4.125	Q3	15
8	American Journal of Clinical Nutrition	27	34.78	8.162	Q1	17
9	Gynecological Endocrinology	27	8.33	2.277	Q3	9
10	Clinical Nutrition	23	13.39	7.643	Q1	10
11	Fertility and Sterility	22	34.68	7.49	Q1	14
12	Scientific Reports	22	11.68	4.996	Q2	9
13	European Journal of Clinical Nutrition	21	21.95	4.884	Q2	9
14	Journal of Nutrition	21	13.76	4.687	Q2	8
15	American Journal of Reproductive Immunology	17	10.59	3.777	Q2	9

ACI, average citations per item; IF, impact factors; Q, quartile in the category; h, h-index.

As shown in Table 4, the top three disciplines according to the number of publications were nutrition dietetics (393, 21.50%), obstetrics gynecology (384, 21.01%), and endocrinology metabolism (337, 18.44%). Additional disciplines represented in the literature were reproductive biology (142, 7.77%), pediatrics (119, 6.51%), biochemistry molecular biology (110, 6.02%), medicine general internal (105, 5.74%), public environmental occupational health (97, 5.31%), immunology (96, 5.25%), multidisciplinary sciences (94, 5.14%), and other disciplines.

**TABLE 4:** The top 20 subject categories in the studies of vitamin D and reproductive health.

Rank	WOS categories	Quantity	Percentage
1	Nutrition dietetics	393	21.50%
2	Obstetrics gynecology	384	21.01%
3	Endocrinology metabolism	337	18.44%
4	Reproductive biology	142	7.77%
5	Pediatrics	119	6.51%
6	Biochemistry molecular biology	110	6.02%
7	Medicine general internal	105	5.74%
8	Public environmental occupational health	97	5.31%
9	Immunology	96	5.25%
10	Multidisciplinary sciences	94	5.14%
11	Medicine research experimental	71	3.88%
12	Allergy	46	2.52%
13	Pharmacology pharmacy	39	2.13%
14	Physiology	33	1.81%
15	Cell biology	32	1.75%
16	Agriculture dairy animal science	27	1.48%
17	Psychiatry	25	1.37%
18	Environmental sciences	23	1.26%
19	Food science technology	22	1.20%
20	Andrology	20	1.09%

### Analysis of authors

As shown in Table 5, Hollis BW from the Medical University of South Carolina has the highest number of published articles (33), followed by Wagner CL from the Medical University of South Carolina (30) and Litonjua AA from the University of Rochester (26). The top three authors in terms of ACI values were Hollis BW (71.00), Wagner CL (64.93), and Camargo CA (49.88) from Massachusetts General Hospital. Five of the top 10 authors are from the United States, four are from England, and one is from Canada.

**TABLE 5:** Top 10 authors in the studies of vitamin D and reproductive health.

Rank	Author	Country	Institute	TP	P	ACI	h
1	Hollis BW	United States	Medical University of South Carolina	33	1.81%	71.00	23
2	Wagner CL	United States	Medical University of South Carolina	30	1.64%	64.93	20
3	Litonjua AA	United States	University of Rochester	26	1.42%	40.58	14
4	Cooper C	England	University of Southampton	25	1.37%	38.28	16
5	Harvey NC	England	University of Southampton	24	1.31%	40.29	16
6	Godfrey KM	England	University of Southampton	23	1.26%	35.48	16
7	Weiss ST	United States	Brigham and Women’s Hospital	23	1.26%	34.09	11
8	Roth DE	Canada	University of Toronto	22	1.20%	26.55	12
9	Camargo CA	United States	Massachusetts General Hospital	16	0.88%	49.88	13
10	Hewison M	England	University of Birmingham	16	0.88%	42.50	10

TP, total publications; P, percentage; ACI, average citations per item; h, h-index.

**FIGURE 4:**
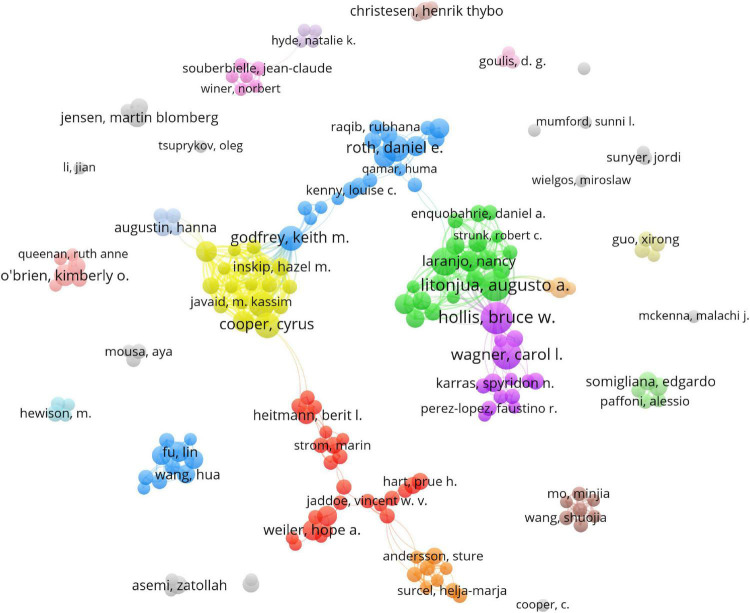
Network map of co-authorship between authors with more than four publications.

We analyzed a total of 204 authors that were co-authored in more than four publications (Figure 4). The five authors with the highest total link strength were Weiss ST (total link strength = 142 times), Litonjua AA (138), Harvey NC (130), Cooper C (129), and Godfrey KM (119). Three of the five are from the same institution called the University of Southampton.

### Citation and co-citation analyses

Table 6 lists the top 10 documents with the highest citations. There were 501 citations for “Vitamin D effects on musculoskeletal health, immunity, autoimmunity, cardiovascular disease, cancer, fertility, pregnancy, dementia and mortality-A review of recent evidence,” (27) followed by “Association between maternal serum 25-hydroxyvitamin D level and pregnancy and neonatal outcomes: systematic review and meta-analysis of observational studies,” (15) with 335 citations. The third-ranked article for the largest number of citations was “Maternal vitamin D status and adverse pregnancy outcomes: a systematic review and meta-analysis,” (14) with 290 citations.

**TABLE 6:** Top 10 citation analysis of documents on vitamin D and reproductive health.

Rank	Title	Journal	Type	Authors	Y	C	IN	CN
1	Vitamin D effects on musculoskeletal health, immunity, autoimmunity, cardiovascular disease, cancer, fertility, pregnancy, dementia and mortality-a review of recent evidence	Autoimmunity Reviews	Review	Pludowski et al. (27)	2013	501	3	1
2	Association between maternal serum 25-hydroxyvitamin D level and pregnancy and neonatal outcomes: systematic review and meta-analysis of observational studies	British Medical Journal	Review	Aghajafari et al. (15)	2013	335	1	1
3	Maternal vitamin D status and adverse pregnancy outcomes: a systematic review and meta-analysis	Journal of Maternal-Fetal & Neonatal Medicine	Review	Wei et al. (14)	2013	290	2	2
4	Vitamin D supplementation for women during pregnancy	Cochrane Database of Systematic Reviews	Review	De-Regil et al. (104)	2012	238	3	2
5	Effect of prenatal supplementation with vitamin D on asthma or recurrent wheezing in offspring by age 3 years the VDAART randomized clinical trial	Journal of the American Medical Association	Article	Litonjua et al. (73)	2016	237	11	2
6	Micronutrient deficiencies in pregnancy worldwide: health effects and prevention	Nature Reviews Endocrinology	Review	Gernand et al. (105)	2016	205	3	1
7	Vitamin D supplementation for women during pregnancy	Cochrane Database of Systematic Reviews	Review	De-Regil et al. (106)	2016	196	4	3
8	Vitamin D and fertility: a systematic review	European Journal of Endocrinology	Review	Lerchbaum et al. (107)	2012	192	1	1
9	Vitamin D supplementation in pregnancy: a systematic review	Health Technology Assessment	Review	Harvey et al. (108)	2014	189	4	1
10	Vitamin D during pregnancy and maternal, neonatal and infant health outcomes: a systematic review and meta-analysis	Pediatric and perinatal epidemiology	Review	Thorne-Lyman et al. (109)	2012	188	1	1

Y, publication year; C, citations; IN, institute number; CN, country number.

Table 7 lists the top 10 references with the highest co-citations. The five references with the largest number of citations were by Aghajafari et al. (15), Holick (28), Holick et al. (29), Hollis et al. (30), and Bodnar et al. (31).

**TABLE 7:** Top 10 co-citation analysis of cited reference on vitamin D and reproductive health.

Rank	Title	Journal	Type	Authors	Y	C	IN	CN
1	Vitamin D deficiency	The New England Journal of Medicine	Review	Holick (28)	2007	381	1	1
2	Evaluation, treatment, and prevention of vitamin D Deficiency: an endocrine society clinical practice guideline	Journal of Clinical Endocrinology & Metabolism	Article	Holick et al. (29)	2011	369	8	3
3	Vitamin D supplementation during pregnancy: double-blind, randomized clinical trial of safety and effectiveness	Journal of Bone and Mineral Research	Article	Hollis et al. (30)	2011	280	1	1
4	Maternal vitamin D deficiency increases the risk of preeclampsia	Journal of Clinical Endocrinology & Metabolism	Article	Bodnar et al. (31)	2007	249	3	1
5	Association between maternal serum 25-hydroxyvitamin D level and pregnancy and neonatal outcomes: systematic review and meta-analysis of observational studies	British Medical Journal	Review	Aghajafari et al. (15)	2013	201	1	1
6	Maternal vitamin D status during pregnancy and childhood bone mass at age 9 years: a longitudinal study	Lancet	Article	Javaid et al. (110)	2006	183	2	1
7	Maternal vitamin D status and adverse pregnancy outcomes: a systematic review and meta-analysis	Journal of Maternal-Fetal & Neonatal Medicine	Review	Wei et al. (14)	2013	180	2	2
8	Maternal vitamin D status during pregnancy and child outcomes	European Journal of Clinical Nutrition	Article	Gale et al. (111)	2008	172	1	1
9	High prevalence of vitamin D insufficiency in black and white pregnant women residing in the northern United States and their neonates	Journal of Nutrition	Article	Bodnar et al. (31)	2007	157	2	1
10	Maternal early pregnancy vitamin D status in relation to fetal and neonatal growth: results of the multi-ethnic Amsterdam born children and their development cohort	British Journal of Nutrition	Article	Leffelaar et al. (112)	2010	139	3	1

Y, publication year; C, citations; IN, institute number; CN, country number.

### Research hotspots and frontier analysis

Keywords are highly summarized and focused on descriptions of the subject of the article, which means that keywords with high frequency can reflect the research hotspots and trends of major issues in related fields. As shown in Table 8, in addition to vitamin D, keywords with a high frequency of occurrence were vitamin D deficiency (890), pregnancy (858), risk (493), vitamin D supplementation (396), 25-hydroxyvitamin D (378), and women (377).

**TABLE 8:** The top 20 keywords in the studies of vitamin D and reproductive health.

Rank	Keywords	Occurrences	Total link strength	Rank	Keywords	Occurrences	Total link strength
1	Vitamin D	912	5,584	11	Outcomes	204	1,501
2	Vitamin D deficiency	890	5,724	12	Preeclampsia	196	1,369
3	Pregnancy	858	5,369	13	Insulin resistance	172	1,122
4	Risk	493	3,317	14	Expression	166	881
5	Vitamin D supplementation	396	2,805	15	Vitamin D receptor	162	876
6	25-hydroxyvitamin D	378	2,613	16	Children	161	999
7	Women	377	2,436	17	Prevalence	153	1,005
8	Association	288	1,929	18	Supplementation	146	1,012
9	Health	213	1,438	19	Pregnant women	144	986
10	Calcium	205	1,261	20	Meta-analysis	135	991

**FIGURE 5:**
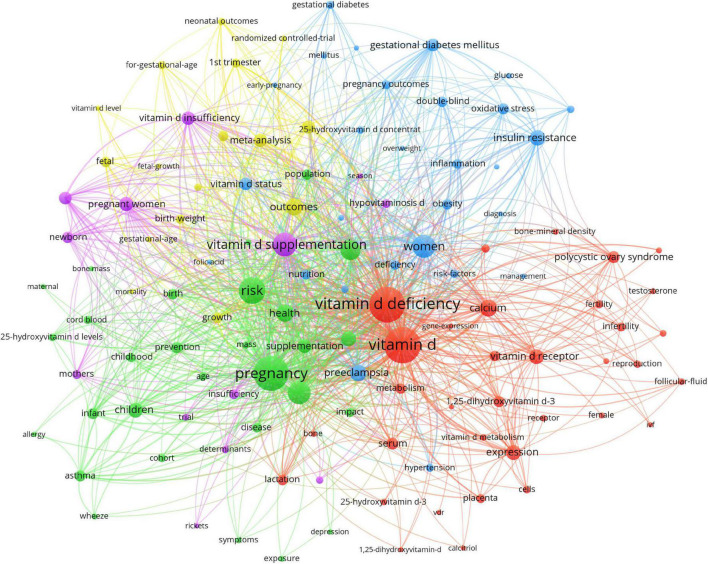
Network visualization of keywords in studies on vitamin D and reproductive health.

We analyzed a total of 120 keywords that were identified as occurring more than 20 times (Figure 5). The size of the node represents the number of times the keyword appears, and the thickness of the curve between the nodes represents the frequency of the two keywords appearing together. The keywords formed five clusters, which represented the five major research directions in the field.

The red cluster was dominated by female, infertility, semen quality, PCOS, and VDR. Vitamin D could affect the synthesis of human hormones (32), the development of oocytes in women (13), the quality of sperm in men (33), and the implantation of the embryo (34). PCOS is one of the critical causes of infertility in childbearing women and its incidence was closely linked to vitamin D deficiency. VDR polymorphisms played an important role in the development of PCOS and associated hormonal and metabolic abnormalities (35).

The yellow cluster focused on randomized controlled trial (RCT), meta-analysis, and neonatal outcomes. RCTs and meta-analyses were the more common types of studies investigating the association between vitamin D and reproductive health. Most RCTs evaluated the efficacy of vitamin D supplementation on IVF outcomes (36), infant acute respiratory infections (37), psychological distress (38), maternal postnatal bone indices (39), and so on. The meta-analysis was a secondary analysis primarily based on RCTs. Neonatal outcomes involved in exploring the effects of maternal vitamin D deficiency on offspring, including small-for-gestational-age (40), neonatal bone mass (41), infant glucose metabolism (42), infant atopic dermatitis (43), infant acute respiratory infections (37), infant birth weight (44), offspring sex ratio (45), infant gut microbiota (46), offspring socioemotional development (47), infant neurodevelopment (48, 49).

The blue cluster was mainly composed of diet, nutrition, and pregnancy outcomes. Vitamin D is an exogenous nutrient that can be influenced by dietary patterns (50), making diet and nutrition a significant subject in this field. Pregnancy outcomes are primarily concerned with adverse maternal effects of abnormal vitamin D status (insufficiency or deficiency), including GDM, hypertension, and preeclampsia (14).

The main research topics of the green cluster were allergy, asthma, wheeze, population, and depression. The first three parts were mainly to study the relationship between preconception and pregnancy serum vitamin D concentration and childhood atopic diseases (51–53). Population referred to discussing the relevant social factors affecting vitamin D levels (54). Depression discussed the role of poor vitamin D in perinatal depression (55).

The high-frequency ones in the purple cluster were vitamin D supplementation, season, and determinants. This clustering mainly addressed relevant determinants of vitamin D levels, such as vitamin D supplementation and season.

**FIGURE 6:**
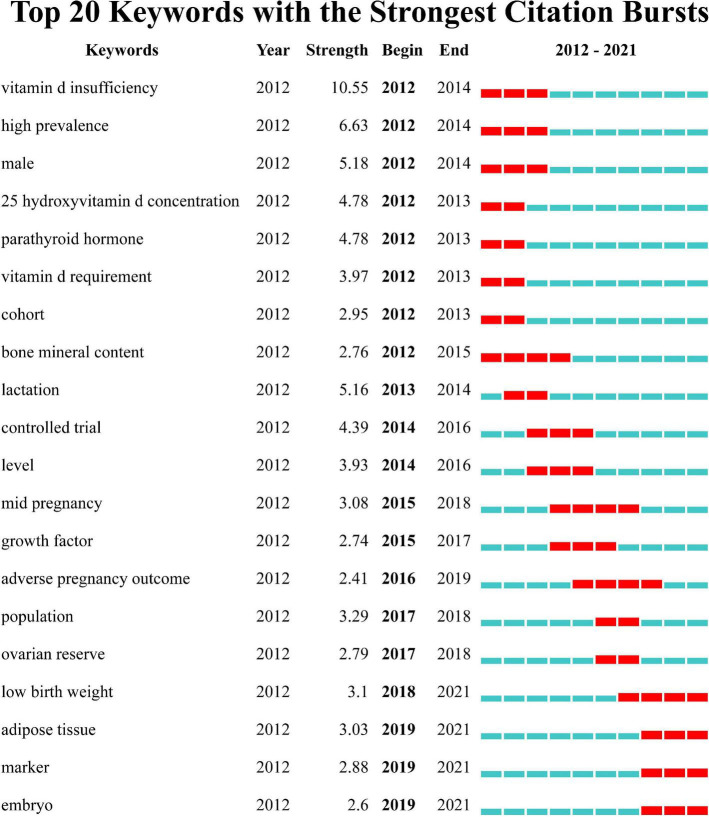
Top 20 keywords with the strongest citation bursts based on CiteSpace.

Burst patterns of keywords can reveal the frontiers and priorities of research between vitamin D and reproductive health. As shown in Figure 6, the timeline is depicted as a year-sliced blue line, where the red section is the detected burst, indicating the start and end years and duration of the burst. Vitamin D insufficiency ranked first with the highest burst strength (10.55), followed by high prevalence (6.63), male (5.18), lactation (5.16), 25 hydroxyvitamin D concentration (4.78), and parathyroid hormone (4.78). The burst times of the terms bone mineral content, adverse pregnancy outcome, and low birth weight lasted for 4 years. Low birth weight, adipose tissue, marker, and embryo are currently within the burst period and might become new research foci in this field.

## Discussion

In this paper, we conducted a bibliometric analysis of the literature on vitamin D and reproductive health published from 2012 to 2021 based on the visualization software CiteSpace and VOSviewer. The spatial and temporal distribution, author and journal contribution, core literature, research hotspots, and research frontier analysis were assessed. The timeline of keywords revealed the evolution of the research theme and the burst analysis suggested current research hotspots.

The research on vitamin D in reproductive health showed a steady upward trend. Research on vitamin D in the field of reproductive health can still arouse great interest so far. On one hand, it is inextricably linked to the benefits of vitamin D. Vitamin D has a physiological role in ovarian follicular development and luteinization, including alteration of AMH signaling, FSH sensitivity, and progesterone production and release (56). Vitamin D also performed vital functions in male reproduction. Serum 25-(OH)D_3_ level was positively associated with sperm motility and VDR in human spermatozoa could be regarded as a positive predictor of sperm quality (57). On the other hand, some of the effects of vitamin D on reproductive health are controversial and have not yet been elucidated. For example, there were differing views on whether vitamin D was associated with IVF outcomes or whether vitamin D supplementation could improve IVF pregnancy outcomes. A retrospective study that analyzed the association between serum 25-(OH)D_3_ level 7 days prior to embryo transfer and pregnancy revealed that vitamin D deficiency impaired clinical pregnancy rate in a single blastocyst transfer cycle (58). Conversely, a RCT by Somigliana et al. showed that a single oral dose of 600,000 IU of vitamin D3 did not improve IVF clinical pregnancy rates in women with normal weight (36).

The study of vitamin D in the reproductive health field showed a pattern of extensive coverage, but uneven development. As shown in Figure 1, approximately more than half of the countries in the world, covering 5 continents, have been involved in this area of research in the last decade. This was probably due to the high prevalence of vitamin D abnormalities (vitamin D insufficiency and deficiency) among the reproductive-aged and pregnant women, which was a worldwide phenomenon. Even in abundant sunshine’s tropical countries like India, the prevalence of vitamin D deficiency among reproductive-aged women is as high as 80%. Numerous studies indicated that vitamin D insufficiency or deficiency varied with geography, economic situation, social status, national policy (food fortification), and individual awareness (59–62). From raising population self-awareness, national intervention to the actual improvement of vitamin D levels, each country needs to further explore its own solutions.

Overall, the America, Europe, Asia, and Oceania were relatively active in this field, while Africa might be in a low active state due to limited economic development. Of these, North America, especially the United States and Canada, probably hold a stronger basis and more mature research system in the field, as 7 and 6 of the top 10 productive institutions and authors come from these two countries, respectively. The Medical University of South Carolina in the United States had the highest ACI, and the top two productive authors are from this institution (Tables 1, 2). The research team mainly focused on prenatal vitamin D screening (63), and the effects of vitamin D supplementation on early life (pregnancy, lactation, and childhood), including glycemic, lipidemic, oxidative stress biomarkers (64), inflammatory biomarkers (65), growth factors, immune-mediators (66), bone mineral density (67), the vaginal microbiome (68), preeclampsia (69) during pregnancy, preterm birth (70), offspring epigenetic clock (71), asthma, allergies and recurrent wheeze (72–75). The conclusions of most original studies were derived from RCTs, with high-level evidence and instructive significance. Furthermore, they also dabbled in basic research, using model animals to explore the effects of a low vitamin D diet on maternal hypertension as well as placental and fetal development (76). Researchers of the University of Southampton focused more on the association between vitamin D and bone-related outcomes such as maternal osteoporosis (39, 77), offspring bone structure (78), bone formation (79), bone mass (80), bone health (81–83) and fractures in late childhood (84). Concurrently, genetic variation in gene expression also received greater interest, with studies showing that detection of genetic susceptibility-related variant genes for vitamin D deficiency may guide vitamin D supplementation (85–87). Europe and Asia are also undergoing rapid development and are represented by the University of Southampton and Tehran University of Medical Sciences, respectively. Tehran University of Medical Sciences paid more attention to the role of vitamin D in male reproduction (88–90) and the mechanism by which vitamin D improved female reproductive disorders including endometriosis (91, 92), PCOS (93, 94), repeated implantation failure (51, 95), and endometritis (96).

It formed a development landscape of multidisciplinary intersection with nutrition, obstetrics and gynecology, and endocrinology metabolism as the core in this field. From Tables 3, 4, we can note that nutrition, gynecology and obstetrics, and endocrinology metabolism are the most dominant WoS categories and the scope of the top 15 journals. These were reflected in the literature on topics such as diet and vitamin D supplementation, the effects of vitamin D on the maternal and offspring, and the expression, role, and variation of vitamin D-related receptors and enzymes. Meanwhile, we should also take note of public environmental, environmental sciences, immunology, multidisciplinary sciences, biochemistry molecular biology, medicine research experimental, pharmacology, cell biology, and so on (Table 4). Multidisciplinary intersection contributed to a more comprehensive understanding of the role of vitamin D in reproductive health to guide the prevention, treatment, and even reversal of the consequences of vitamin D deficiency. The participation of basic disciplines is to split the whole into the tissue, cellular, and molecular levels, to gain an in-depth understanding of the mechanism.

Risk factors and adverse impacts of vitamin D deficiency were the focus of current research. The effect of maternal vitamin D levels on fetal lipid metabolism and the prediction of fertility by vitamin D-related markers might be hot spots and frontier areas in the field. Adipose tissue serves as a reservoir for vitamin D and affects its activity (97). Maternal vitamin D deficiency may affect fetal weight by influencing lipid metabolism, but the current findings are controversial. A birth cohort-based study in China suggested that maternal serum 25-(OH)D_3_ was positively associated with fetal birth weight and that vitamin D deficiency during pregnancy increased the risk of low birth weight infants (98). A study performed by Morales et al. indicated that maternal deficit of 25(OH)D_3_ was associated with an increased risk of fetal overweight and overweight in offspring at age 1 year (99). This might be due to maternal vitamin D deficiency resulting in polarization in the adipose depots (100). Moreover, vitamin D-related indexes are expected to be new markers for fertility assessment. Li et al. reported urine VDBP levels were significantly positively correlated with ovarian reserve and were expected to be a biomarker for predicting ovarian reserve (101). In assisted reproductive technology, vitamin D level in the follicular fluid could be used as a marker of oocyte quality, and vitamin D level in serum could be used as a marker of *in vitro* fertilization outcome (102). For men of childbearing age, the vitamin D metabolizing enzyme CYP24A1 was positively correlated with total sperm count, concentration, motility, and morphology, and the expression of CYP24A1 at the annulus of human spermatozoa might serve as a novel marker of semen quality (103). Therefore, further research is needed to clarify the above-mentioned effects of vitamin D, and RCT with a higher level of evidence might be a more meaningful research approach.

To our knowledge, this study is the first bibliometric analysis to explore the research on vitamin D in reproductive health. However, it had a few limitations. First, we only selected the WoS database and were limited by visual analysis software to include only the WoSCC. Second, we only analyzed the literature over the last decade, but this field has developed for such a considerable period of time that some previous views might be ignored by us. Finally, some of the more recently published articles may have been overlooked by the hotspot analysis because they did not have sufficient citations.

## Conclusion

Vitamin D holds great research significance and the clinical application potential in the field of reproductive health. Based on visual analysis software, we evaluated the evolution process, contribution distribution, development trend, research hotspots, and frontiers of vitamin D research in reproductive health over the past decade. The field has received great attention globally, and the multidisciplinary intersection is the development trend. Furthermore, the effect of maternal vitamin D levels on fetal lipid metabolism and the prediction of fertility by vitamin D-related markers might be the focus of the research, and the exploration of population heterogeneity in the diagnostic criteria of vitamin D deficiency and the specific mechanisms of vitamin D effects on reproductive health should also be paid attention to.

## Data availability statement

The datasets used and/or analyzed during the current study are available from the corresponding author on reasonable request.

## Author contributions

XZ and YL were responsible for experiment conception and design, collection and assembly of data, data analysis and interpretation, and manuscript writing. SW and SZ contributed acquisition data and revised the manuscript. JT designed the work, provided technical guidance, and finally approved the manuscript. All authors read and approved the final manuscript.

## Footnotes

1
https://www.mapchart.net/
